# A narrative analysis of women's experiences of planning a vaginal birth after caesarean (VBAC) in Australia using critical feminist theory

**DOI:** 10.1186/s12884-019-2297-4

**Published:** 2019-04-29

**Authors:** Hazel Keedle, Virginia Schmied, Elaine Burns, Hannah Grace Dahlen

**Affiliations:** School of Nursing and Midwifery, Western Sydney University, Locked Bag 1797, Penrith, NSW 2751 Australia

**Keywords:** Australia, Vaginal birth after caesarean (VBAC), Qualitative, Narrative analysis, Feminist research, Power relationships

## Abstract

**Background:**

Most women who have a caesarean can safely have a vaginal birth after caesarean (VBAC) for their next birth, but more women have an elective repeat caesarean than a VBAC.

**Methods:**

The aim of this qualitative study was to explore the experiences of women planning a vaginal birth after caesarean (VBAC) in Australia, the interactions with their health care providers and their thoughts, feelings and experiences after an antenatal appointment and following the birth. The study explored the effect of different models of care on women’s relationships with their health care provider using a feminist theoretical lens. Eleven women who had previously experienced a caesarean section and were planning a VBAC in their current pregnancy used the ‘myVBACapp’ to record their thoughts after their antenatal appointments and were followed up with in-depth interviews in the postnatal period.

**Results:**

Fifty-three antenatal logs and eleven postnatal interviews were obtained over a period of eight months in 2017. Women accessed a variety of models of care. The four contextual factors found to influence whether a woman felt resolved after having a VBAC or repeat caesarean were: ‘having confidence in themselves and in their health care providers’, ‘having control’, ‘having a supportive relationship with a health care provider’ and ‘staying active in labour’.

**Conclusions:**

The findings highlight that when women have high feelings of control and confidence; have a supportive continual relationship with a health care provider; and are able to have an active labour; it can result in feelings of resolution, regardless of mode of birth. Women’s sense of control and confidence can be undermined through the impact of paternalistic and patriarchal maternity systems by maintaining women’s subordination and lack of control within the system. Women planning a VBAC want confident, skilled, care providers who can support them to feel in control and confident throughout the birthing process. Continuity of care (CoC) provides a supportive relationship which some women in this study found beneficial when planning a VBAC.

**Electronic supplementary material:**

The online version of this article (10.1186/s12884-019-2297-4) contains supplementary material, which is available to authorized users.

## Background

Vaginal birth after caesarean (VBAC) is a safe mode of birth for the majority of women [1], however, rates remain low in many parts of the world. In Australia 14% of women had a VBAC in 2016 [2]. Across Europe VBAC rates vary from 20 to 55% [3, 4]. In the US the national average VBAC rate in 2016 was 12% [5]. Research suggests at least 45% of women plan to have a VBAC for their next birth [6].

Women planning a VBAC, in Australia, can access antenatal care through a variety of models, dependent on access, availability, resources and choice. Models of care may include: standard antenatal care, which in most instances is fragmented, women may see different midwives and/or doctors in a hospital clinic and this separates the woman’s journey into antenatal, birth and postnatal episodes of care; midwifery group practice (MGP), where women receive continuity of care (CoC) from the same midwife or small team of midwives based in hospital; GP shared care, where women see their General Practitioner (GP) and providers in a hospital; private obstetric care with a private obstetrician; and privately practising midwives (PPM) [7]. Not all models are available across Australia and not all models will accept women planning a VBAC, resulting in many women having the limited option of standard maternity care [8]. It is estimated that only 8% of women have access to CoC with a midwife in Australia [8]. Not all MGPs will accept women who have had a previous caesarean section and many birth centres have this as an exclusion criteria due to the risk of uterine rupture and the subsequent identification of women planning a VBAC as ‘high risk’ [9].

Women planning a VBAC have shown a clear preference for CoC with a midwife [10, 11]. Limited research exists around the benefits for women planning a VBAC and accessing CoC with a midwife. A small study from China has found that women receiving CoC with a midwife experienced shorter labours and higher VBAC rates [12]. Martin et al. (2014) evaluated a next birth after caesarean clinic (NBAC) in Western Australia and found that women accessing the antenatal clinic had increased knowledge about VBAC compared to women not attending the clinic [13]. A study exploring midwives and obstetricians views on caring for women planning a VBAC emphasised the need for CoC yet highlighted the restrictions for women planning a VBAC and accessing MGP models of care in a system that is often not supportive of women’s choices [14].

Several researchers have conducted qualitative studies of women’s experiences of VBAC. These studies tend to either focus on women during pregnancy to explore decision making or contacting women postnatally to retrospectively explore women’s’ experiences of having (or not having) a VBAC [15, 16].

The aim of this qualitative study was to explore the experiences of women planning a VBAC in Australia, the interactions with their health care providers and their thoughts, feelings and experiences after an antenatal appointment and following the birth. The study also explored the effect of different models of care on women’s relationships with their health care provider using a feminist theoretical lens.

The results presented in this paper are from the qualitative phase of a three-phased sequential exploratory mixed methods study. The first phase was a meta-ethnography exploring women’s experiences of VBAC [10]. The overarching theme of ‘the journey from pain to power’ identified the transformative journey women embarked on from their previous caesarean through to their VBAC. The importance of this journey, for women planning a VBAC, influenced the focus for the second phase of the study (the one reported in this paper) which is a qualitative focus on the experiences of women when planning a VBAC across different models of care and their thoughts, feelings and experiences after an antenatal appointment and following the birth. Phase three, which will follow this study, will be an Australian survey of women planning or having experienced a VBAC, designed from the findings of phase one and two, along with questions generated from previous research undertaken by this team [17].

## Methods

Critical feminist theory provided the lens to inform all aspects of this study, from the research question, data collection, data analysis and interpretation.

### Critical feminist theory

Critical feminist theory merges concepts from critical theory and feminist theory [18, 19]. Critical theory focuses on identifying inequalities due to class, race, industrial relations and globalisation [19] and feminist theory brings a primary focus on inequalities due to gender. Both theories acknowledge the difficulties in implementing change in existing institutional structures and aim to elicit system change [19, 20]. Critical feminist theory acknowledges the many levels that influence women’s experience from a gendered and power perspective by highlighting the hegemonic effect patriarchy has on the experiences of women [20, 21].

Feminist researchers have identified the issues of power in the technocratic model of birth where authoritative knowledge is held by expert clinicians and pregnancy and birth are medicalised to fit into the biomedical norms of increasingly restrictive ‘textbook births’ including not going ‘overdue’ and reaching 10cms within an acceptable timeframe [22–25]. Biomedical models of health care privilege male dominated obstetrics over female dominated midwifery often silencing midwifery or subordinating midwives to medicine and doctors [26, 27]. Alternatives to hierarchical models include midwifery continuity models of care where women and midwives work in partnership; midwives provide woman-centred care with an increased level of agency afforded to women; and midwives are positioned as equals within the multidisciplinary health care team [28]. This study explores women’s experiences of seeking a VBAC across models of care and explores the effect of these models of care on women’s relationships with their health care professional (HCP) from a feminist viewpoint.

Having a VBAC is difficult to achieve in the current medicalised system. Critical feminist theory can deepen understanding about the experiences of women planning a VBAC by exposing the systematic changes required for women to have a respectful and equitable VBAC journey [18]. Research conducted with a feminist lens can impact social change and highlight inequalities for women [29].

### Participants

Participants were recruited through social media groups that focused on pregnancy, birth and VBAC. A flier was posted on relevant online sites such as VBAC Australia Support Group and local pregnancy groups with information on the study. When potential participants contacted the researcher for further information they were forwarded a participant information sheet and a link to an information video explaining the study. Exclusion criteria included: women without a history of a previous caesarean, not currently pregnant, not residing in Australia and non-English speaking women. Eleven women were recruited and completed the study.

### Data collection

A purpose-built app was designed, by the first author, to fulfil all the needs of data collection. The design and evaluation of the ‘myVBACapp’ is described in detail in a previous paper [30]. Using the ‘myVBACapp’ allowed for pregnant women (with a smartphone) across Australia, to access the study. Women were able to provide real-time accounts of their thoughts, feelings and experiences after an antenatal appointment in the form of an audio or video recording and this minimised issues of retrospective reporting or filtering and analysing effects of written responses [30]. This form of data collection aligned with feminist theory principles as the participant decided when and what to record and whether or not to send the recording to the researcher [18].

Women were required to download the app from their iOS or Android App Stores and register their details on the app. The app contained the consent form for the study. Once the app was registered and activated on their smartphone, the participants recorded a three-minute audio or video log. The information video contained details and suggestions on when to record the logs which would preferably be within 24 h of the antenatal appointment. As the women were recording their thoughts and experiences, and not the specifics of their interaction with an HCP, their HCP did not need to be aware that the participant was involved in the study. Women were encouraged to refrain from mentioning the names of HCP, or facilities, in their recordings. Any accidental mention of HCP or hospitals was deleted from the data and not reported. In total 53 logs were recorded by 11 women, with logs posted from 14 weeks gestation to immediately post birth. The logs were transcribed by the first author.

Semi-structured interviews were conducted with all women six weeks after birth, either face to face in a location convenient to the participant, or on the phone, dependent on their location and availability. Interviews ranged from 45 to 90 min in length. The interviews were undertaken by the first researcher recorded and then transcribed by an external transcriber. Interview questions can be found in Table 1. Due to the previous information shared by the women in the antenatal logs, the researcher personalised some questions to clarify issues initially mentioned in the logs. This strategy ensured all relevant concerns or experiences were explored.

**Table 1:** Interview questions

Interview questions	
1	Tell me about the support you received for your birth choice
2	What do you think was helpful / unhelpful in the support?
3	Tell me about your labour and birth
4	How did you feel after your birth?
5	What would you advise women who are planning a VBAC?

### Narrative analysis

Narrative analysis was used to analyse the women’s stories, using story telling as the basis for exploration of a phenomenon [31, 32]. The narrative includes both the obvious story that is told and the underlying influences that frame the narrative [33]. Stories can be sequential and structured using timelines during analysis [34]. First, the individual stories of the participants were analysed as a whole, and compared against other complete stories, until saturation was achieved [31]. During this process the common and dominant themes were identified across the stories alongside experiences that were in contrast to the dominant themes. Although data collection included an online approach the participants appeared to build a relationship with the first author through directing their recorded story to the researcher. Narrative researchers recognise the integral role they play as audience to the participant and acknowledge the impact of this relationship on the data [32]. Narrative analysis is appropriate within a critical feminist framework due to the importance of storytelling and privileging the ‘voices’ of the individual participants [35].

The steps taken are listed in the analysis process Table 2. The women’s stories comprised data from both their sequential transcribed antenatal logs and their postnatal interviews. Authors read the woman’s stories and regular team meetings occurred throughout the research process to confirm preliminary and ongoing analysis and to confirm or discuss the resulting themes.

**Table 2:** Analysis process

Step	Analysis process
1	Reading through women’s stories making initial notes
2	Second read through, making notes and producing timeline for stories
3	Writing a summary for each story
4	Comparing stories for similar themes
5	Formalising contextual factors as a research team
6	Marking the impact of each factor for each participant

### Reflexivity

Reflexivity is important for both critical feminist research and to increase validity and transparency in qualitative research [36]. All four authors are midwives with the first author being both a woman who had experienced a VBAC and a midwifery researcher and PhD candidate. To recognise this position as an insider, field notes were maintained to journal the researcher’s thoughts and feelings. Notes were made after listening to antenatal logs and after postnatal interviews and included contextual information such as gestation and the HCP as well as researcher observations and thoughts. Field notes informed the analysis through reflection and discussion with the research team.

### Consolidated criteria for reporting qualitative studies (COREQ)

In accordance with BioMed Central editorial policies the qualitative methods and the reporting of results adhere to the COREQ guidelines [37].

## Results

Eleven women completed the study. Their ages ranged from 30 to 39 years and they were all either married or in a long-term relationship. The highest level of education ranged from one woman finishing Year 12 at high school, one woman completing to diploma level, two women to undergraduate degree level and six women completing postgraduate qualifications. Eight women had a combined yearly household income of more than $100,000 Australian Dollars (AUD) and three women ranged from $40,000–$90,000 AUD. Nine women were born in Australia, one woman in New Zealand and one woman was from Chile. There was one woman who identified as having an Aboriginal and/or Torres Strait Islander heritage. Five women were living in city locations and six women in regional locations across the Australian States of New South Wales, Queensland and Victoria. Four women had a private obstetrician, two women had a PPM, three women accessed MGP, one woman saw a GP trained in Obstetrics and one woman attended antenatal clinic.

Five women had a VBAC, one had an instrumental birth and five women had a repeat emergency caesarean. All names are pseudonyms. Table 3 gives further description about the models of care the women in this study accessed.

**Table 3:** Models of care participants accessed

Models of care used in this study	Abbreviation	Description
Private Obstetrician	Priv Obst	Private specialist doctor providing antenatal and birthing services
Privately Practising Midwife	PPM	Private midwife providing continuity of care. Can provide home birthing services or have visiting rights in a hospital.
Midwifery group practice	MGP	Midwife working as part of a group providing continuity of care in a hospital environment
General Practitioner with obstetric training	GP/Obst	GP with obstetric training who provides antenatal care and birthing services
Clinic	Clinic	Doctor or Midwife providing antenatal appointments in hospital

When analysing the women’s stories, four main contextual factors emerged that were important to whether women felt resolved, or disappointed, after either having a VBAC or following a repeat caesarean. Feeling resolved can be defined as ‘to settle or find a solution to a problem or contentious matter’ [38]. Feeling resolved also encompasses how experiencing a VBAC assisted women in settling the question of their ability to give birth vaginally. For women who had a repeat caesarean and felt resolved they were also able to gain knowledge about their previous experience.

**Fig. 1:**
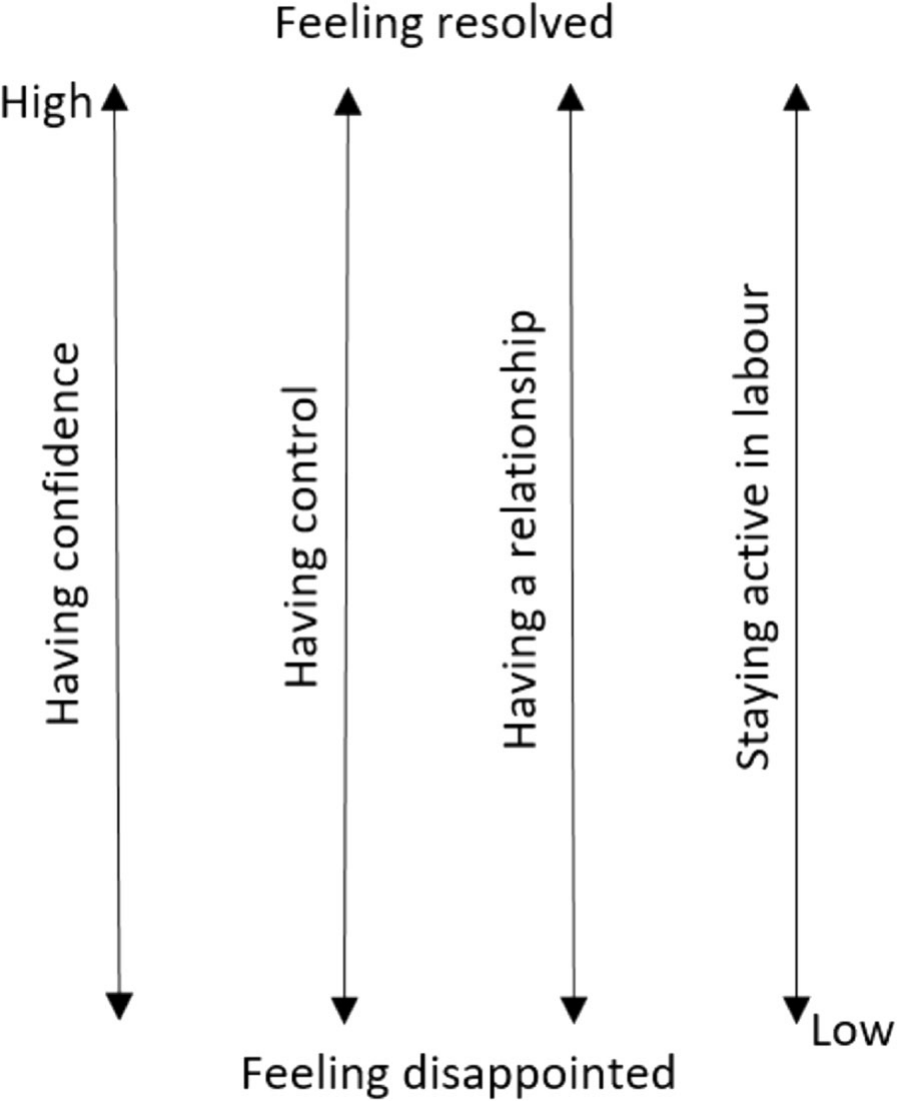
Four contextual factors which influenced women’s experience when seeking a VBAC

The four contextual factors identified were: having confidence in themselves and in their HCPs; having control; having a supportive relationship with HCPs; and staying active in labour. Figure 1 shows the four contextual factors with arrows that indicate the woman could be high or low on that factor.

The four contextual factors worked independently from each other but had a cumulative effect. For example, having high confidence, having a high sense of control, a strong relationship and an active labour resulted in the woman feeling resolved after her birth experience, regardless of mode of birth. Both Arabelle, and Angela, had a repeat emergency caesarean but the cumulative positive effect of the four factors resulted in them feeling resolved after their caesareans. Bianca and Isabella had experienced high levels of all four factors and felt resolved after their VBAC.

Exemplars of each of the factors, having confidence in themselves and in their HCPs, having control, having a supportive relationship with HCPs and staying active in labour, are explicated below by presenting various individual women’s stories, as identified in Table 5. Each of the stories is presented with a timeline to demonstrate when, in the antenatal logs, this part of their story occurred. Although there were 11 women in the study, due to the length and depth of each story, only four of the individual stories are shared in full in this paper. The individual stories were chosen as their story highlighted the issues in each of the factors. Timelines for all the women in the study are available in Additional file 1 and the individual stories will be in the PhD thesis. An explanation of each factor is given before the story and quotes from other participants are used to demonstrate the extent / strength to which they factor within these women’s stories and the names of these women are listed under illustrative date in Table 4. After each quote is the pseudonym of the woman, the timing of the quote (e.g. 33/40 is 33 weeks gestation, PN is postnatal) and model of care.

**Table 4:** Women’s stories

Factor	Women’s story	Illustrative data from other participants
Having control	Tonia	Arabelle, Carley
Having confidence	Isabella	Bianca, Emma, Abbey
Having a relationship	Bianca	Calista, Jemima
Having active labour	Dehlia	Carley, Bianca, Angela

### Having control

Women who felt that their wishes and choices were respected felt more in control of their pregnancy, birthing decisions and outcomes. Women such as Bianca, Arabelle, and Dehlia demonstrated high levels of feeling in control in their narratives, and these women also experienced having a relationship with one lead HCP.

Feeling in control impacted how Arabelle felt after a repeat emergency caesarean. Arabelle had CoC with a MGP midwife and went into spontaneous labour after her waters broke at 39 weeks.

*“My body started pushing but I still had no urge to push myself, which was kind of weird. But after 4 hours of that I then said it’s not going to happen. Like if it ends up in surgery, I’m completely fine with that.” (Arabelle, PN, MGP)*

Postnatally, Arabelle reflected on how in control she felt and the benefit of support from a midwife she knew, *“because I think apart from that I was in control the whole way. There was at no point somebody said to me, “No, you can’t do that, … I think the continuity of care, having this same midwife for every single appointment, she stayed with me from the moment I laboured until I went to recovery and so that made a huge difference.” (Arabelle, PN, MGP).*

Neither Carley or Emma demonstrated feeling in control of their choices or outcomes. Once Carley knew her planned homebirth after caesarean (HBAC) was going to be a planned induction of labour in hospital she *“just cried the whole Sunday” (Carley, PN, PPM)* because *“I knew if I was being induced, that was it, it was not going to work.” (Carley, PN, PPM)* Carley had a Foleys catheter inserted to induce labour overnight and the next day had her membranes ruptured. When labour didn’t ‘progress’ she requested synthetic oxytocin but was refused by the obstetric team. When reflecting on how she was treated in labour she felt that by not being given options she was denied control of her situation.

*“You need to give people choices. You need to give people options. And we weren’t, at any point in that whole scene, we were not near a situation where you would, you know, an emergency that overrules everything.” (Carley, PN, PPM)*

#### Tonia

**Fig. 2:**
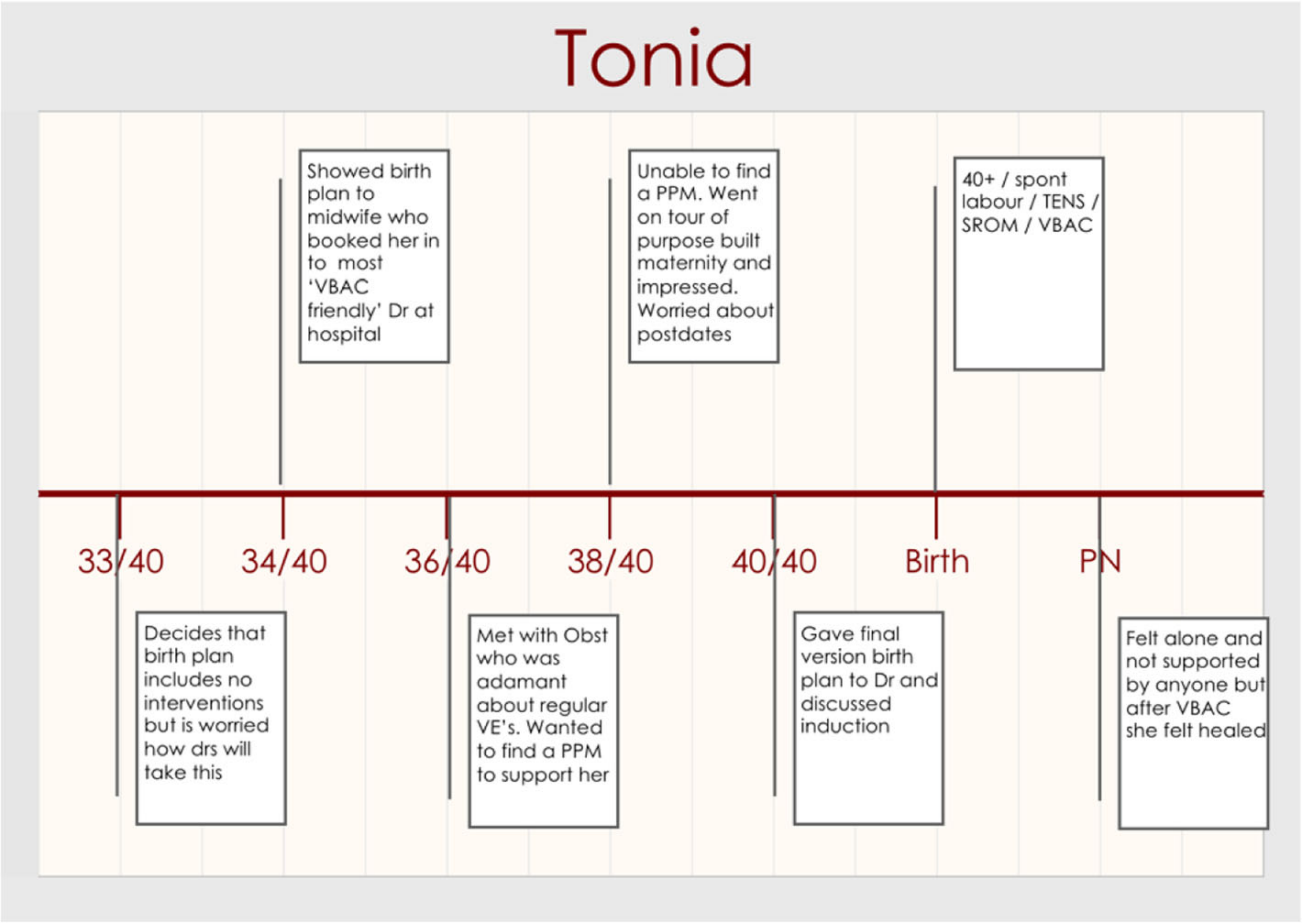
Tonia Timeline

The narrative which best captures the struggle to maintain control is Tonia’s story. Tonia’s story represented a continual struggle between maintaining control for herself and being controlled by the hospital system. Tonia had experienced CoC with a midwife for her first birth through an MGP model and birth centre, but she was unable to access the same model due to it excluding VBAC, therefore she accessed standard antenatal care. Figure 2 shows the timeline of Tonia’s story.

Tonia felt that her last birth was traumatic, and she wanted to retain control throughout this pregnancy and birth. One way she decided to do this was by limiting the interventions required by the VBAC hospital guidelines / policy. Tonia wrote a birth plan highlighting her wishes for the upcoming labour and birth, and as demonstrated in Fig. 2, she reflects on this in her 33 week recording.

*“So, my 34-week appointment which is coming up next week is to go through my birth plan, which I’m a little bit apprehensive about because I’m declining all of their recommendations for admission which is an IV on arrival, continuous fetal monitoring, a VE on admission and every 4 hours after that. I don’t want to be strapped to the bed; I don't want to have an IV in my arm. I’m declining the VE cos they were excruciating in my last labour and they didn't help, they made everything worse and I just, I’m not going to have a bar of it. Yeah, so, we’ll see how that goes.” (Tonia, 33/40, clinic)*

**Fig. 3:**
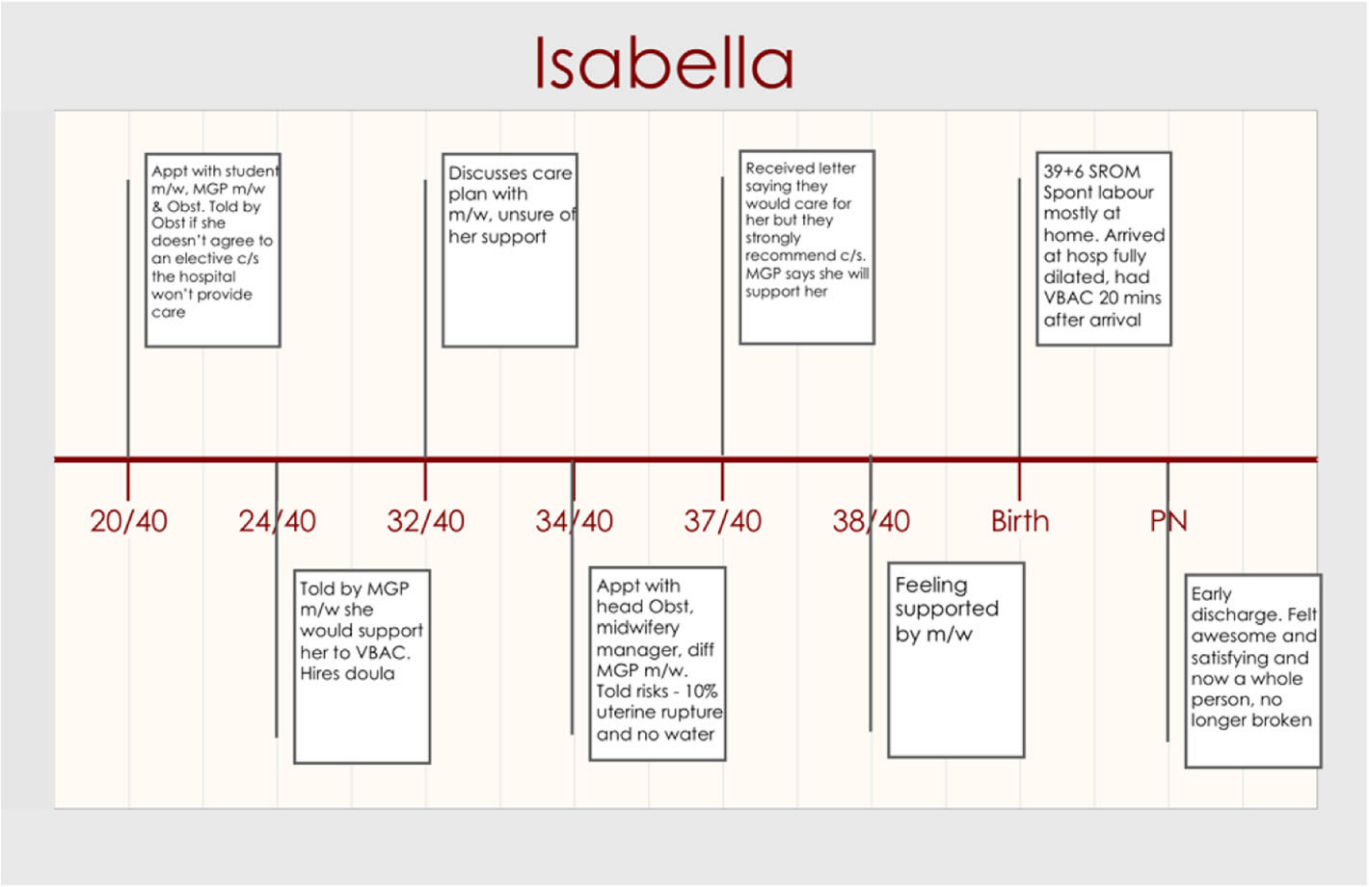
Isabella Timeline

After presenting this to a midwife at 34 weeks, as seen in Fig. 3, Tonia was referred to a ‘VBAC friendly’ doctor at the hospital. Tonia reported back that this next appointment went *“spectacularly badly” (Tonia, 36/40, clinic)*. Tonia described how inflexible the doctor was. *“The OB was quite adamant that these* [vaginal examinations] *were essential and that they had to be done and that there were no other ways to assess progress and they wouldn’t know what to do without them.” (Tonia, 36/40, clinic).* Tonia then explained in the log why she was declining vaginal examinations.

*“I’m not declining them for fun, I’m declining them because I find them excruciating and triggering of past rapes, I’ve been raped numerous times and I just do not want VE’s because being triggered in the middle of labour will probably stall my labour and I just, yeah well, I’m not going to agree to them. Not unless I feel safe and comfortable and having the threat of that looming overhead will make it difficult for me to feel safe and comfortable.” (Tonia, 36/40, clinic)*

Following this appointment Tonia felt that the doctors were not willing to compromise or relinquish any of their control for her individual benefit.

“*So, it’s almost as if they’re treating a bag of statistics rather than an individual because that’s what it feels like, it doesn’t feel like they’re looking at the whole picture, doesn’t feel like they’re looking at me as a patient, they’re just looking at me as like another collection of statistics.” (Tonia, 36/40, clinic)*

As seen in Fig. 2, Tonia tried to hire a PPM but due to her late gestation was unable to find a midwife with vacancies and she continued to receive antenatal care through the hospital clinic. Tonia started labouring at 40 + 1 weeks and went into the hospital. Although Tonia requested no internal examinations she felt manipulated into an examination by an obstetrician when her midwife *“went to lunch” (Tonia, PN, clinic)*. Tonia was in the vulnerable position of being in labour and the doctor used their position of power to coerce her.

With only the use of a transcutaneous electrical nerve simulation (TENS) machine in labour, Tonia went on to birth her baby *“she tumbled out of me and I picked her up and put her to my chest. She was purple and crying. And I was crying. It was beautiful, and I couldn’t believe it.” (Tonia, PN, clinic).*

Overall Tonia felt that her journey to having a VBAC was a fight, with her fighting against the system alone with no-one on her side.

*“I knew that I would have to fight for this. Like that became really clear that the hospital system, although it’s a public health system, it’s not one that treats you as an individual. And if I wanted to have this VBAC I was going to have to fight for it.” (Tonia, PN, clinic)*

### Having confidence

The confidence women had in their own ability to have a VBAC was evident throughout their antenatal logs. Women such as Isabella, Calista, Tonia and Bianca did not doubt their ability to have a vaginal birth and had a strong confidence in the birthing process, despite their previous caesarean experience.

*“I’m feeling still pretty confident even though getting a little bit nervous I guess cos it’s starting to get towards labour time, buy yeah, still confident with my little team!” (Bianca, 37/40, PPM)*

Alongside their own confidence to have a VBAC, women observed and commented on the confidence they believed their provider had in their ability to have a VBAC. As no HCPs were included in this research it is important to clarify that this is the woman’s perception of how confident their HCP was in her capacity to have a VBAC. Women reflected on comments or actions of their HCP to gauge their confidence. Comments such as *“Oh yeah, we’re attempting a VBAC, OK we’ll have to see how we go, if all the stars align then that should be ok” (Emma, 36/40, GP/Obst)* and *“She thinks it will be an elective caeser or an induction if I’m lucky” (Abbey,37/40, PrivObst)* made these women doubt the level of support and confidence their HCP had in their ability to have a VBAC.

In comparison, Bianca reflected that her midwife had confidence and belief in her ability to VBAC: *“I found it really encouraging that she’s, she agrees with me and she’s confident as well that I can get a relatively medicine free VBAC which is awesome because you don’t often get that from many other places, so that made me feel a bit better.” (Bianca, 38/40, PPM)*

#### Isabella

Isabella’s narrative best reflected the impact of confidence on her ability to have a VBAC, despite strong opposition. Although many of her experiences show aspects of intimidating and controlling behaviour from HCPs, her story shows her steadfast determination and the confidence her MGP midwife demonstrated when supporting Isabella in her birth choices. Figure 3 shows Isabella’s timeline.

Isabella was planning a VBAC, however she had an added complicating factor of an inverted T scar from her previous caesarean, where a vertical incision is extended in the midline of a transverse lower segment incision forming an inverted T-shaped incision [39]*.* She was aware of the recommendations from the state health guidelines *“the policies for the hospital and [State] Health, they recommend a repeat caesarean at 37 weeks for future pregnancies on this incision.” (Isabella, 20/40, MGP).*

Isabella accessed CoC with a midwife through the hospital MGP program and a midwifery student who was following Isabella throughout her pregnancy and birth care. Early on in her pregnancy, as demonstrated in Fig. 3, Isabella found out the opinions of the obstetric team regarding her birth choice when a consultant obstetrician stated, “*If I didn’t agree to a repeat caesarean at 37 weeks that the hospital, which is a public hospital, would not provide care for me for this pregnancy.” (Isabella,20/40, Clinic Dr).*

Although Isabella felt *“particularly upset and shocked and concerned” (Isabella, 20/40, MGP)* following these comments she didn’t change her plans for a VBAC and hired a doula for extra support.

Whilst being aware of the opinions of the obstetric team, Isabella tried to ascertain the confidence and feelings of her midwife regarding her choice of having a VBAC. Her trust and confidence in her midwife grew during the pregnancy. At 24 weeks gestation, as seen on Fig. 3, her midwife stated *“no matter what I decide to do … that the midwife team is there to support me, even if they don’t agree with what I choose to do” (Isabella, 24/40, MGP).* By 38 weeks Isabella reflected *“I have established a pretty good relationship with my midwife and confident with her support with me and my choices for this birth” (Isabella, 38/40, MGP).*

Although Isabella was confident with her midwife’s support she continued to get opposition from the obstetric team. At 34 weeks she attended a meeting with an obstetrician and the midwifery team where she was given statistics that contradicted the research she had read regarding uterine rupture. The appointment ended after the obstetrician asked a very emotive question; *“she said to me how would I feel if I had a hysterectomy and dead baby as a result of my choices?” (Isabella,34/40, MGP)*. Isabella confidently responded with *“I just said that I would feel more comfortable if I made choices that aligned with me and what I believe is right rather than doing something that I don’t believe is right” (Isabella,34/40, MGP).*

Isabella went into labour spontaneously, laboured at home and arrived at the hospital in the second stage of labour, birthing within 20 min of arrival. Her doula commented afterwards about the midwife’s ability to buffer and protect Isabella’s birthing space from unnecessary personnel entering the room.

*“My doula said to me like how awesome it was to see my midwife keep everybody away from me and let me do what I needed to do. She thought that was just amazing to see her do that because I guess she hasn’t seen other midwives doing that for the women before.” (Isabella, PN, MGP)*

Following her VBAC Isabella reflected *“It was like I feel awesome that I did it, but I never questioned my ability to do it … without any interventions, without any pain medication, without anything I knew I could do it” (Isabella, PN, MGP).* The benefits for Isabella were on her ability to bond better with her baby and how she felt about her own emotions around her body.

*“This time I feel like a whole person. I don’t feel like a shattered mirror trying to put everything back together. Like I feel like I’m in the best place that I can be to look after a new little baby and my family and myself.” (Isabella, PN, MGP)*

### Having a relationship

Having a relationship describes women who formed a respectful and supportive relationship with their HCP and had CoC. The relationship became a partnership where women and their HCP had equity and decisions were made together. Women who demonstrated a strong relationship with their HCP included Bianca, Calista, Arabelle, Isabella, Jemima and Dehlia. Four women had CoC with a midwife and two had CoC with a private obstetrician.

The support differed depending on the type of care providers. With midwifery CoC women reported more chatting, more time at appointments and more access outside of appointments, as well as support throughout the labour and birth experience. With the four women who had a private obstetrician the women reported the appointments as shorter, less chatty and women would not know who their midwife would be during labour. Calista recorded how she could access her MGP midwife when she needed to discuss her concerns.

*“I didn’t really have an appointment with my midwife as such, but I’ve been in contact with her quite a bit over the last two days and I’ve spoken to her for over ½ an hour today so in my eyes it’s pretty close to what you would do in an appointment anyway.” (Calista, 38/40, MGP)*

Women were aware of conflicting opinions and attitudes between the obstetric and midwifery teams. After Calista had her VBAC her midwife informed her that before she went into a labour *“a couple of the obstetricians in town had already written off to my midwife that I wasn’t going to be able to deliver a VBAC and that I would be in for a c-section and one of them had joked with her, oh I’ll see you in theatre” (Calista, PN, MGP).* This made Calista reflect on how important her midwife was in buffering the negative attitudes around her.

*“And then as I came to realise after, they buffer for you from a lot of the information that you don’t need to know about at the time.” (Calista, PN, MGP)*

Jemima changed private obstetricians for this following pregnancy and was able to reflect on the differences between the two practitioners. Jemima found this obstetrician *“100% supportive of my decision” (Jemima, PN, PrivObst)* and encouraging when she had doubts.

*“Just getting to the end of this pregnancy, I was tired and grumpy, he kind of got me back on track. Obviously, during the labour, he did the same thing. I just felt like really respected by the second obstetrician.” (Jemima, PN, PrivObst)*

#### Bianca

**Fig. 4:**
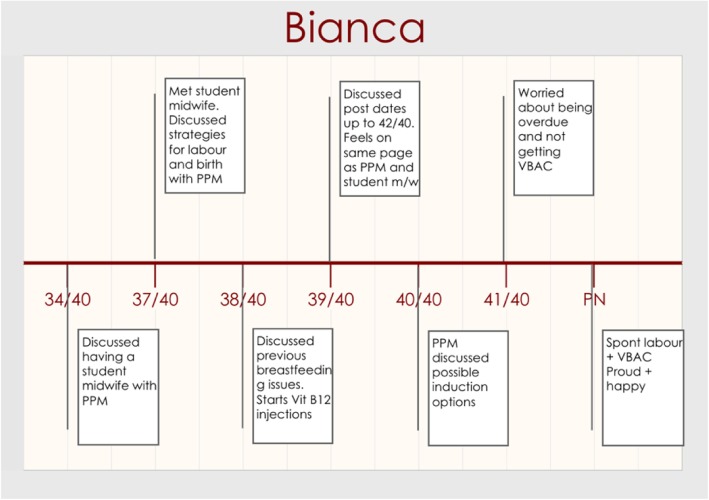
Bianca Timeline

The narrative that shows the benefit of a good relationship with HCPs is Bianca’s. Bianca accessed a PPM with visiting rights to assist her to have a VBAC in hospital and during her logs and interview described the strong relationship based care she received from her midwife. Bianca’s timeline is shown in Fig. 4.

Bianca found her midwife *“very calming” (Bianca, 37/40, PPM).* As she approached her due date at 39/40, as seen on Fig. 4, she reported on how she had discussed going overdue with her PPM. She described how:

*“We are both happy to go over if everything is looking good, to go over the 40 weeks but that will depend on how much fluid and movement and making sure that everything looks good and that [PPM] is happy to do that as well. I do really respect her decisions and I don’t think that she would ever do anything that is not in my best interest.” (Bianca, 39/40, PPM).*

Bianca explained at 40/40 how she could negotiate her decisions and options with her midwife.

*“She really encouraged me to own my decisions and not be coerced into something that doesn’t seem right and that includes things that she recommends as well. She’s always given me all of the available options and then let me choose from there. After giving me all of the information and giving me time to research by myself.” (Bianca, 40/40, PPM)*

Bianca went into spontaneous labour and her midwife spent time with her at home before they transferred to the hospital. She used the shower in hospital and within five hours she was pushing and birthed her baby. After her VBAC she felt *“I’m really quite proud that I did it because so many people did doubt me” (Bianca, PN, PPM)*.

In the postnatal interview Bianca was asked about how supportive her midwife was regarding VBAC and Bianca interestingly related the midwife’s own birthing experiences to how supportive and encouraging the midwife was.

*Interviewer: “Did you find her very supportive about your wanting to have a VBAC?” Bianca: “Yes. She's actually had one of both so she kind of understood the desire for it. Yes, she was really supportive, like any time I had any concerns she just made it totally disappear. She was always really encouraging and was happy to go with whatever I wanted to do as well, which was good. I never felt pressured into anything. And yeah, she provided a lot of the support through information really and just being realistic and nice with everything.” (Bianca, PN, PPM)*

Bianca reported no examples of needing to liaise with other HCPs which potentially suggests the benefits of a PPM with visiting rights to a hospital with no requirement to involve obstetric care unless needed in an emergency. This made Bianca’s care appear streamlined and consistent and contrasts strongly with Carley who had a PPM but without visiting rights.

### Staying active in labour

This study shows that labour and birth was the pivotal climax in the women’s journey to planning a VBAC. How women were treated and how active they could remain had ramifications on their ability to feel in control of their choices for labour and birth and in their confidence that they had the best chance to have a VBAC. Confidence, control and relationships were all tested during labour and birth. The meticulous planning during pregnancy on how to cope in labour affected the women’s confidence in her ability to continue or to remain in control if she had interventions such as regional anaesthesia. Carley felt that *“I don’t feel like a fighter, I feel like I had that epidural when I could have hung out more, you know” (Carley, PN, PPM).*

Women who had reduced interventions in labour and remained mobile and upright for as long as possible felt this positively contributed to having a VBAC or feeling resolved after a repeat emergency caesarean. Strategies women used in labour to stay active included using the shower, the exercise ball, sterile water injections, standing, and using a TENS machine. Bianca described how *“I had hot water like fall off on my back and that was really it. I kind of just used water for the most part, water and quietness” (Bianca, PN, PPM).*

Being able to remain active for as long as possible helped Angela feel resolved with her repeat emergency caesarean. Angela felt that *“I literally tried everything. I feel like if I … so the whole time I was like, oh, what if I did this in my first birth? What if I did that? I tried everything I wanted to try that time this time and obviously there was no changing the outcome. So, I did what I could and it still happened the same way. So obviously, there’s nothing more I could have tried” (Angela, PN, Priv Obst).*

#### Dehlia

Dehlia’s story shows the importance of remaining active and how she was encouraged to do this through the supportive relationship she had with her private obstetrician and the midwife allocated to her in labour.

**Fig. 5:**
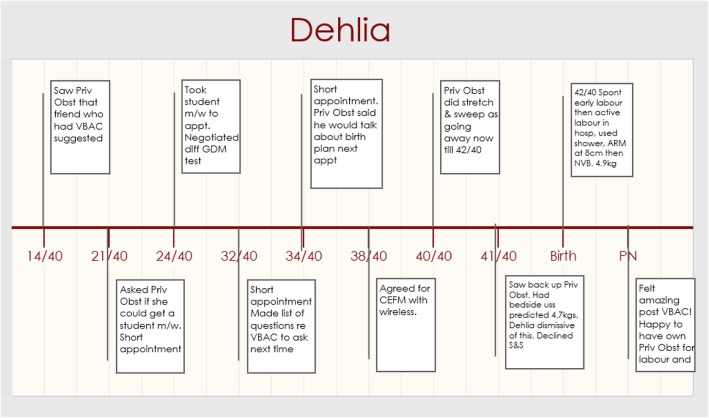
Dehlia Timeline

Dehlia chose her private obstetrician because *“one of my friends recently had a VBAC with him so hoping he’ll be supportive the whole way through” (Dehlia, 14/40, Priv Obst).* Figure 5 shows the timeline of Dehlia’s story.

Although regular measurements were done in pregnancy, Dehlia reported that her doctor did not focus on the size of the baby and that she also felt he believed in her ability to have a VBAC. Dehlia’s timeline (Fig. 5) shows that although the appointments weren’t ‘chatty’, she was able to negotiate different interventions. The timeline also shows she saw a different obstetrician at 41 weeks as her obstetrician was away, and she dismissed the results of a bedside ultrasound predicting a large baby and didn’t go into labour until her obstetrician was back at 42 weeks.

During labour Dehlia found she had a supportive midwife on duty and her obstetrician was also there. *“And the midwife who was on then was someone that I recognised from my first birth, and she recognised me as well. And just her manner, I was a lot more comfortable with her and she’s more willing to make compromises and things like that. The labour continued really well from then on … And when my obstetrician was there, he was … yeah, I felt really comfortable with him as well.” (Dehlia, PN, Priv Obst).*

Dehlia laboured in the shower and surprised herself as she held off from having an epidural. She remained mobile and upright throughout her labour and had a VBAC. Dehlia ‘s first baby was 3.8kgs so everyone was surprised when she birthed a much larger baby who was 4.9kgs!

*“She was born at the same size as my first one when she was two months old. I still can’t believe it!” (Dehlia, PN, Priv Obst)*

Upon reflection Dehlia recognised the impact staying active in labour had on her ability to have a VBAC *“I’m sure that if I had an epidural, there’s no way I would have been able to push her out, I think I’m pretty sure I would have ended up in a caesarean.” (Dehlia, PN, Priv Obst).*

## Discussion

This study provides a unique insight into the experiences of women planning a VBAC in Australia. Using an app to obtain thoughts and feelings after each antenatal appointment allowed for collection of real time data. The influence of the individual factors: control, confidence, relationship and active labour, had an impact on the women’s journeys when planning a VBAC.

These four contextual factors were not mutually exclusive and often interacted during the women’s journey. This discussion will explore the intricacies of the four factors reported in this study alongside relevant research.

### Control

Women described the experience of ‘having control’ as being an important component of their pregnancy and birth experience. Women planning a VBAC had a desire for control over their choices, wishes and outcomes, influenced by the perceived lack of control experienced during their previous caesarean and their understandings of what behaviours or interventions influenced their feelings of control. In this study having control was experienced on a continuum with women such as Bianca and Dehlia displaying more control than Carley or Emma.

Many feminist authors have argued that since the rise of the technocratic patriarchal model of obstetric care, female bodies have been viewed as ‘broken’ if not fitting within set biomedical norms [23], with the authoritative knowledge around birth held by experts instead of women [25]. This authoritative knowledge was evident with Abbey who stated how her obstetrician didn’t believe in her ability to have a VBAC. It was evident that the majority of participants were seeking control over their birthing experience, however their ability, or lack of ability, to have control differed amongst women. Control is related to power relationships and consequently feeling out of control can reflect patriarchal power exerted over women and their bodies during pregnancy and childbirth [23, 40] and influencing decision making around VBAC [41].

The relationship between control and power is complex and this complexity is explicated in Foucault’s (1980) concept of knowledge / power [42]. In this understanding there are opportunities for resistance however the biomedical model has become dominant, promoted by obstetricians and some midwives and permeates all levels of society including consumers and the media [43–45]. This dominance results in ‘disciplinary power’ which can appear inconspicuous and part of everyday culture until faced with opposition [44]. When both Tonia and Isabella resisted the dominant medical discourse they experienced punishment, due to their perceived non-compliance when HCPs used distressing language and threatened to withdraw care.

Fahy & Parratt (2006) expand on the Foucauldian ideas of power/knowledge to explore the ‘terrain’ of the birthing room and the power relationships within the room, which they titled ‘jurisdiction’ [44, 46]. Jurisdiction is comprised of the core aspects of integrative / disintegrative power and midwifery guardianship/ domination that both occur across a continuum. At one end of the continuum, power will be vested in the woman and she will be supported in her birth choices and the other end of the continuum the power has been removed from the woman and practices done to the woman [44, 46]. In this study the four factors all occurred along a continuum. Drawing on Fahy & Parratt’s (2006) work, at the positive end of the continuum the woman has been supported and guarded by the midwife to freely respond and react to her labouring body and instinctive feelings and at the negative end of the continuum, the woman feels undermined and unable to exert her own power. In this context, women were denied control and left disappointed with potentially another traumatic birthing experience. Carley was a good example of having control during her pregnancy but during her induction of labour feeling out of control and being left feeling disappointed with her treatment and lack of options.

Women can feel out of control in many ways, through enforced interventions and expectations to fit a biomedical norm, through HCPs withholding medical information, a lack of adequate monitoring and from obstetric violence [23, 47]. Obstetric violence includes dehumanisation of women during pregnancy and childbirth and the loss of autonomy and involvement in decision making of women due to the medicalisation and pathologisation of natural processes [40, 47]. Examples evident in this study included the restrictive effects of policies and procedures at the hospital level enforced by HCP, such as restriction of VBAC services, pressure to be continuously monitored and have vaginal examinations and the language used (such as ‘not allowed’).

Feeling out of control during the labour and birth process is a common experience for women who have identified as having experienced birth trauma [48–50], including women with a previous caesarean [10]. In this study women who felt in control during their VBAC journey felt this contributed to feeling resolved postnatally. Feeling in control related to experiencing respectful maternity care and a willingness from HCPs to listen and negotiate with women about their wishes and choices during pregnancy and birth. Findings from this study support the findings of Rhodes (2013) and Thompson & Downe (2010) who showed that for women with a history of a traumatic birth, feeling supported and respected in the subsequent pregnancy can result in feelings of resolution and experiencing a ‘redemptive birth’ regardless of mode of birth [51].

### Confidence

Having confidence helped women in this study have agency and maintain control. Confidence has been defined as the “emotional basis of action and agency” [52] and is necessary for the individual to stand apart and take their own path. Women who were perceived to have high confidence in this study had a belief in their ability to have a vaginal birth and most were supported in their belief by their HCP. Studies into other aspects of maternity care have shown that women may choose an out of hospital birth, for example, if they have confidence in their ability to birth naturally [17, 53] and if they have confidence in their midwives’ expertise and practice [54]. Maternity care providers in America identified that midwifery CoC and a woman-centred approach can increase women’s confidence during birth [55]. Research identifies that having a previous vaginal birth increases the rates of a successful VBAC [56], potentially related to the confidence women may have gained in their birthing ability.

Women who have had a previous caesarean may have reduced confidence in their ability to give birth [24, 57] and this can be further reduced by feelings of subordination and objectification both within the birthing room and within society [23, 47]. The women in this study who had a lack of confidence indicated this through narratives demonstrating apprehension and uncertainty, often mirroring comments from their HCPs. Women’s narratives identified the importance of feeling that their HCP was also confident in the woman’s ability to have a VBAC. Nilsson et al. (2015) explored the views from women who had experienced a VBAC in countries with high VBAC rates and found that HCPs who demonstrated confidence in their knowledge and experience of VBAC assisted women to feel calm and safe and in turn increased women’s self-confidence.

### Relationship

In this study a strong relationship between the woman and her HCP was demonstrated through feelings of support and respect and enhanced by CoC. HCPs had the ability to directly influence whether a woman felt resolved or disappointed through providing support or giving disrespectful care during pregnancy and labour and birth. Kennedy’s work on exemplary midwifery practice, showed that HCPs had the ability to become the instruments of supportive care [58] or interventions hindering the way for women on their birthing journey. When midwives are working alongside women in a partnership model there is the opportunity to ameliorate the patriarchal hierarchal model and focus on relationships based on equality and respect [25, 59]. The women in this study who described supportive CoC relationships found their HCP respected the women’s knowledge and decisions.

Experiencing respectful supportive care from a HCP and from a supportive health care system increases women’s satisfaction with their childbirth experience [60]. To encourage this a systemic approach, alongside an individual approach, is needed. Health care systems need to support evidence based models of care, such as midwifery CoC, as these increase women’s satisfaction [61]. To be supportive, HCPs need to feel confident and secure in their position. This includes receiving appropriate work and pay conditions [62] to prevent HCPs from experiencing burnout [63]. When HCPs feel undervalued this in turn affects their ability to give optimum care to women [64].

### Active labour

All women in this study said they wanted to stay active during labour and give themselves the best opportunity to have a VBAC. These findings are echoed in the qualitative interviews from the USA by Hall et al. (2018) who found that women wished for freedom of movement and comfort as strategies for labour and this was also found to increase the probability of normal birth [65, 66].

During labour both Abbey and Carley felt a lack of control when their wishes and desires for an active labour or reduced interventions weren’t respected or acted upon. Policies regulating VBAC practices may result in restricted mobility, rigid time frames and limited access to water immersion [1, 67] which can affect the options women have, as well as the control they feel during the birth. Providing a birthing environment that is designed to optimise the labouring and birthing woman with privacy, comfort, access to water, and space to mobilise, can enhance the woman’s experience and prevent her from feeling fear, as was explored in the sub-concept of ‘terrain’ in the theory of Birth Territory developed by Fahy and Pratt [46].

This study has highlighted the impact that patriarchal maternity systems have on the subordination of women through unequal power relations. Women planning a VBAC benefit from supportive and respectful relationships as these can increase women’s’ confidence in their ability to birth, maintain women’s agency and control and result in feelings of resolution, regardless of mode of birth.

### Limitations and strengths

The eleven participants in this study were all from one country and accessed models of care available in Australia. Models of care differ greatly internationally and will impact on women’s individual experiences. All women were required to speak and read English and have access to technology with a smart phone to download the app, and this excluded women who were non-English speaking and those with limited resources. All the women self-selected and may not be a true representation of everyone planning a VBAC. The women were recruited through social media interest groups which may mean these women had stronger preference for their birth choice.

Strengths of this study included the ability to access data in real time without reliance on retrospective recall. Women could be honest about their experiences in the privacy of their own home and away from their HCP. This also prevented any behaviour modification from the HCPs as they were potentially unaware of the women being on the study. Women also potentially benefited from using reflection as a tool during their pregnancy and one woman noted that if she was to be pregnant again she would use a similar process as this study for those positive benefits. Using the ‘myVBACapp’ gave the opportunity for women across Australia, in both city and regional locations, to participate in the study, allowing for a greater representation across a variety of locations.

## Conclusion

This study followed women throughout their pregnancy journey with snapshots of their thoughts and experiences recorded on the ‘myVBACapp’. Critical feminist theory was used as the lens through which, the difficult journeys some of the women experienced can be identified. Issues such as going against policy or declining vaginal examination and interventions highlight the systemic belief in expert-driven care as more important than individualised consumer-driven care alongside the notion of silencing the ‘difficult woman’ who speaks out. The positive influence that midwifery continuity of care brings to these systemic patriarchal issues was evident with the women in this study that experienced it and further studies exploring such models of care for women planning a VBAC is necessary and important.

Four contextual factors identified in this study influenced whether a woman felt ‘resolved’ after the birth experience - having confidence in themselves and in their HCP, having control, having a supportive relationship with an HCP and staying active in labour. A supportive and respectful relationship and continuity of care with an HCP was pivotal for women planning a VBAC and to feeling resolved after their birthing experience.

## Additional file

Additional file 1: Timelines word document. Timelines for women. Word document with seven timelines of women in study (DOCX 135 kb)

## Abbreviations

CoC: continuity of care
GP: General practitioner
HBAC: Homebirth after caesarean
HCP: Health care professional
MGP: Midwifery group practice
PN: Postnatal
PPM: Privately practising midwife
TENS: Transcutaneous electrical nerve simulation
VBAC: Vaginal birth after caesarean
