# Supplementary material for: A narrative analysis of women's experiences of planning a vaginal birth after caesarean (VBAC) in Australia using critical feminist theory
Source: BMC Pregnancy Childbirth. 2019 Apr 29;19:142. doi: 10.1186/s12884-019-2297-4 (PMC6489285; doi:10.1186/s12884-019-2297-4)

Supplementary information

Abbey Timeline


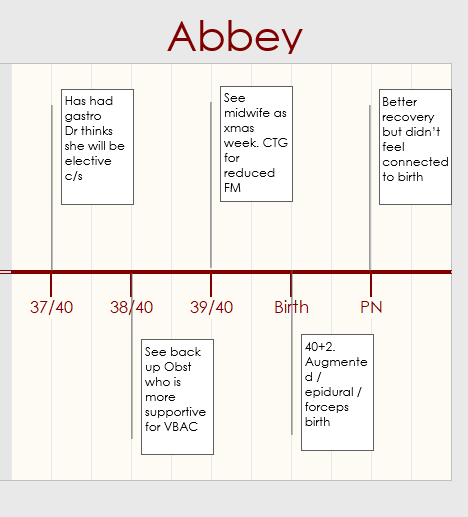


Angela Timeline


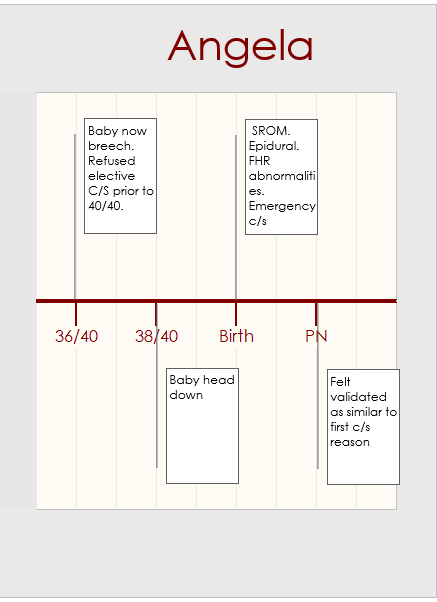


Arabelle Timeline


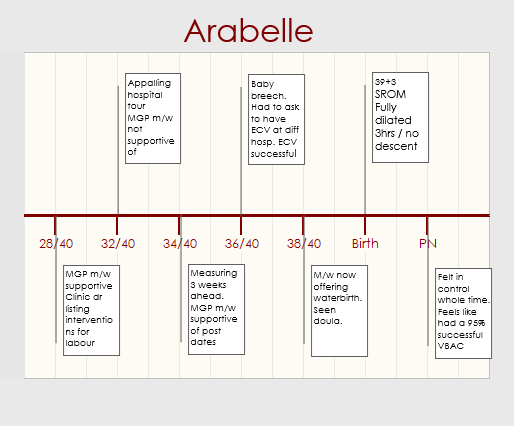


Calista Timeline


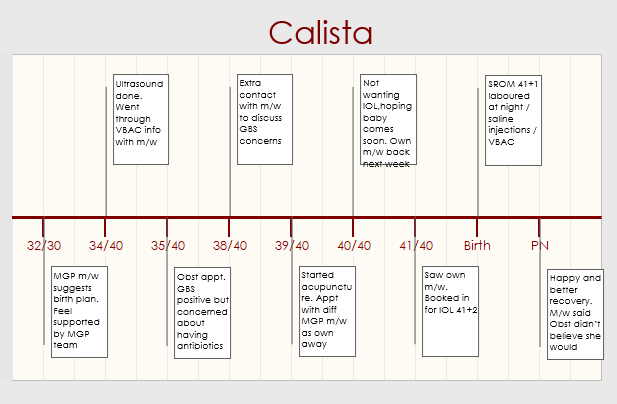


Carley Timeline


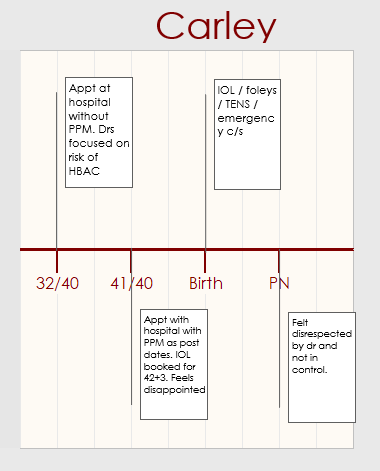


Emma Timeline


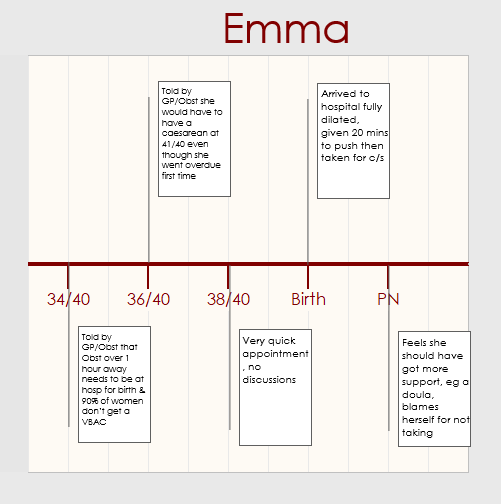


Jemima Timeline


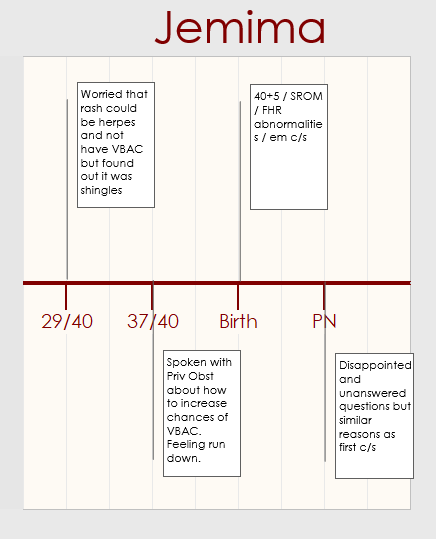

Supplement: Supplementary file 1 — Timelines word document. Timelines for women. Word document with seven timelines of women in study (DOCX 135 kb) [file 12884_2019_2297_MOESM1_ESM.docx]
